# Mammographic density in birth cohorts of Danish women: a longitudinal study

**DOI:** 10.1186/1471-2407-13-409

**Published:** 2013-09-05

**Authors:** Sophie Sell Hellmann, Elsebeth Lynge, Walter Schwartz, Ilse Vejborg, Sisse Helle Njor

**Affiliations:** 1 Department of Public Health, University of Copenhagen, Copenhagen, Denmark; 2 Mammography Screening Unit, Odense University Hospital, Odense, Denmark; 3 Diagnostic Imaging, Copenhagen University Hospital, Copenhagen, Denmark

## Abstract

**Background:**

Breast cancer is the leading malignant disease among western women with incidence increasing over time. High mammographic density is a well-established risk factor for breast cancer. We explored trends in mammographic density across birth cohorts to gain further insight into possible time trends in women’s mammographic density that might explain the historical increase in breast cancer incidence.

**Methods:**

Data derived from two mammography screening programs in Denmark from 1991 to 2001, including on average 41,091 women from Copenhagen and 52,938 women from Funen aged 50–69. Mammographic density was assessed qualitatively (fatty or mixed/dense) by senior screening radiologists. The proportion of women with mixed/dense mammographic density was calculated by age at screening, screening period, and birth cohort. The Generalized Estimating Equations were used to calculate odds ratios and 95% confidence intervals. All statistical tests were two-sided.

**Results:**

The proportion of women with mixed/dense mammographic density increased from 45% among women born in the 1920s to 75-80% among women born in the 1940s. In Copenhagen, the age-adjusted odds ratio (95% CI) of mixed/dense mammographic density in women born in 1941–42 was 2.48 (2.22-2.76) compared with women born in 1921–22. In Funen, the age-adjusted odds ratio of mixed/dense mammographic density in women born in 1946–47 was 5.89 (5.32-6.51) compared with women born in 1924–25. Hormone use had a greater impact on mammographic density in birth cohorts of the 1920s compared with those of the 1940s.

**Conclusions:**

We found suggestive evidence of a birth cohort pattern in mammographic density and an attenuated impact of hormone use in younger compared with older birth cohorts suggesting that postmenopausal mammographic density could be linked to changing exposures accumulated over time in women’s lives.

## Background

Breast cancer is the leading malignant disease among western women [1]. Denmark has an age-standardised breast cancer incidence of 101.1 per 100.000 [2], with an increased trend across birth cohorts [3-5]. Age and mammographic density are important risk factors for sporadic breast cancer [6]. Mammographic density is defined as the proportion of fibroglandular relative to fatty breast tissue [7-9]. Mammographic density in more than 75% of the breast has been associated with a four- to six fold increased risk of breast cancer compared with less than 5% mammographic density [8,10]. This association between mammographic density and risk of breast cancer has been confirmed by qualitative and quantitative measures of mammographic density [8].

Studies have found a decrease in average mammographic density with increasing age and during menopause [6,11], an inverse association with parity [12] and body mass index [6,13,14], and a positive association with hormone use [14-16]. Women’s mammographic density might vary across birth cohorts by changes in women’s exposures over time. We explored the importance of birth year on women’s mammographic density to investigate the historical increase in breast cancer incidence.

## Methods

### Screening programs

We obtained mammography data from two independent organised population-based screening programs in Denmark conducted at specialised clinics from 1991 to 2001. Mammography screening was implemented in Denmark in the municipality of Copenhagen in April 1991 and in the county of Funen in November 1993. The two programs offered biennial screening free of charge, representing 95,000 women aged 50–69 years [17]. Personal invitation to screening was based on the updated central population register since 1968 including information on personal identification number, historical addresses, emigration, immigration, and vital status for all persons ever residing in Denmark. Women with breast cancer diagnosis were covered by clinical mammography programs.

A two-view mammography, craniocaudal and oblique, was performed at initial screening. Independent double reading by consensus reading was performed by highly trained radiologists, who assessed mammographic density qualitatively into fatty or mixed/dense mammographic density. Fatty mammographic density were equivalent to BI-RADS (Breast Imaging Reporting and Data System (BI-RADS) Atlas, 2008) Density code 1 and part of Density code 2, and mixed/dense mammographic density equivalent to part of BI-RADS Density code 2, Density code 3, and Density code 4. Women with fatty mammographic density and a negative screening were scheduled for a one-view oblique mammography at the subsequent mammography screening. Women with mixed/dense mammographic density and a negative screening were scheduled for a two-view mammography at the subsequent screening. Subsequent mammograms were compared with those from earlier screening. The same procedure was followed throughout the study period [18,19]. The mammography screening programs for Copenhagen and Funen complied with quality performance indicators as specified in European guidelines [18,19].

### Data

Data were collected on women participating in any of the first five invitation rounds in Copenhagen from April 1991 to March 2001, and from Funen any of the first four invitation rounds from November 1993 to December 2001. The analysis was restricted to this period to ensure consistency in the assessment of mammographic density. In Copenhagen, all mammograms were assessed by the same senior radiologist from 1991–1998, supplemented by an added senior radiologist from the autumn of 1996, who by consensus reading assessed all mammograms for the rest of the study period [20]. In Funen, the same senior radiologist assessed all mammograms during the study period. Data contained information on personal identification number, date of examination, mammographic density (fatty or mixed/dense), and screening outcome. Both programs used analog mammography throughout the study period.

The density coding was re-evaluated using data from a Copenhagen study on long-term breast cancer risk in women with false-positive screening test [21]. In total, 118 negative screening mammograms taken prior to the false-positive screening were re-evaluated. Among 31 women with fatty mammographic density, 32% (n=10) had BI-RADS code 1, 61% (n=19) BI-RADS code 2, and 7% (n=2) BI-RADS code 3 at re-evaluation. Among 87 women with mixed/dense mammographic density, 1% (n=1) had BI-RADS code 1, 31% (n=27) BI-RADS code 2, 62% (n=54) BI-RADS code 3, and 6% (n=5) BI-RADS code 4 at re-evaluation. Manual control of 3 misclassified women revealed they were borderline cases with changed density status over time.

We analyzed data available on systemically administered estrogen and combined estrogen-progestogen associated with increased mammographic density [14-16]. Data were obtained from the Odense University Pharmacoepidemiological Database, which contained individual level information on reimbursed prescription drugs purchased in all pharmacies in the city of Odense and suburbs from October 1990, and all of Funen from the end of 1992 [22]. The completeness of the register is high and includes information on personal identification number, date of purchase, drug code, commercial name, and prescribed dose [22]. Never users were defined as women with no registered purchase of hormones prior to screening. Current users were defined as women who purchased a quantity of hormones before attending screening that would last until 14 days before screening. For Copenhagen, data on hormone use was available only for the later part of the study period and was therefore not included in the analysis.

### Statistical analysis

The analysis was restricted to women aged 50–71 years for Copenhagen and 50–69 years for Funen at the time of screening. Copenhagen invited all women aged 50–69 years at the beginning of each invitation round resulting in a higher actual age at screening. Funen invited women aged 50–69 years at the time of invitation. Age, period, and birth cohort were analysed as categorical variables. Age was classified into two-year age groups (50–51, 52-53….70-71). Period was defined by the two-year invitation rounds for Copenhagen (April 1991 to April 1993,…April 1999 to April 2001) and for Funen (January 1994 to December 1995…January 2000 to December 2001) to have periods of equal length. Birth cohort was defined in the diagonals by the linear relation birth cohort = period-age for Copenhagen (1921-1922….1949-1950) and Funen (1924-1925….1948-1949). For each screening program, we constructed a two-way table between two-year age groups and two-year invitation rounds (period). The same tables were made for never and current hormone users from Funen. For simplicity, past users were omitted from this analysis.

The probability of mixed/dense mammographic density was calculated by dividing the total number of screened women with mixed/dense mammographic density by the total number of screened women, and 95% confidence intervals (95% CI) for all probabilities were calculated using the binominal distribution. The association between mammographic density and birth cohort, screening age, and screening period was evaluated within the Generalized Estimating Equation framework. An exchangeable correlation structure was assumed. As age, period, and birth cohort are correlated, only two of these variables were included in each regression model to avoid multicollinearity. The GENMOD procedure (SAS version 9.1) was used to calculate crude and adjusted odds ratios (OR) and 95% confidence intervals (95% CI). Two-sided p-values were calculated at the <0.05 significance level.

The unique personal identification number issued to all residents of Denmark was used for the record linkage. The study was approved by the Danish Data Inspection Agency, which according to Danish legislation serves as ethical approval of register-based research.

## Results

On average 41,091 women were invited to mammography screening during each of the first five screening rounds in Copenhagen (Table 1). Coverage in Copenhagen decreased from 70% to 61% from the first to the fifth screening round. Data on mammographic density was available for 99% of the screened women. In Funen, on average 52,938 women were invited to mammography screening during each of the first four screening rounds (Table 1). Coverage in Funen remained constant at 84% during 1993–2001. Data on mammographic density was available for 98% of the participating women.

**Table 1:** Mammography screening program in the Copenhagen (1991–2001) and Funen (1993–2001) regions, Denmark

**Copenhagen**					
**Screening period, Year**	**1991-93**	**1993-95**	**1995-97**	**1997-99**	**1999-2001**
Target population (TP), N (%)	43092	41159	40037	40304	40865
**Coverage by age (years), N (%)**	29991 (69.6)	25920 (63.0)	24981 (62.4)	25269 (62.7)	24910 (61.0)
50-54	5779 (58.8)	5737 (54.7)	6668 (57.3)	7200 (52.7)	6918 (50.5)
55-59	6967 (72.8)	6279 (67.9)	6127 (66.4)	6699 (70.2)	7215 (67.1)
60-64	7377 (69.2)	5995 (61.4)	5600 (63.2)	5299 (63.9)	5289 (64.2)
65-71^1^	9868 (75.7)	7909 (67.8)	6586 (63.9)	6071 (68.8)	5488 (67.3)
**Data on mammographic density, N (%)**					
Yes	29991 (98.8)	25920 (99.4)	24981 (99.4)	25269 (99.4)	24910 (99.4)
No^2^	363 (1.2)	163 (0.6)	157 (0.6)	149 (0.6)	145 (0.6)
**Mammographic density, N (%)**					
- Fatty	15814 (52.7)	13093 (50.2)	11668 (46.7)	10669 (42.2)	8983 (36.1)
- Mixed/dense	14177 (47.3)	12827 (49.2)	13313 (53.3)	14600 (57.8)	15927 (63.9)
**Funen**					
**Screening period, Year**	**1993-95**	**1996-97**	**1998-99**	**2000-01**	
Target population (TP), N (%)	49666	51985	54198	55905	
**Coverage by age (years), N (%)**	41381 (83.3)	43483 (83.7)	44272 (82.0)	46007 (82.3)	
50-54	12755 (86.4)	15046 (89.5)	15052 (84.8)	14755 (85.3)	
55-59	10813 (87.4)	10869 (84.5)	11554 (83.6)	13033 (83.7)	
60-64	9370 (81.9)	9370 (81.0)	9682 (81.3)	9941 (81.3)	
65-69	8416 (75.7)	8198 (75.5)	7984 (74.5)	8278 (76.7)	
**Data on mammographic density, N (%)**					
Yes	40253 (97.3)	42767 (98.4)	43564 (98.4)	45275 (98.4)	
No^2^	1128 (2.7)	716 (1.6)	708 (1.6)	732 (1.6)	
**Mammographic density, N (%)**					
Fatty	16650 (41.4)	18031 (42.2)	15733 (36.1)	13452 (29.7)	
Mixed/dense	23603 (58.6)	24736 (57.8)	27831 (63.9)	31823 (70.3)	

^1^One woman was aged 72 years at screening in the screening period 1993–95 in Copenhagen. ^2^Women with a positive mammography screening and subsequent histopathology verified ductal carcinoma in situ (DCIS) or invasive breast cancer.

**Figure 1:**
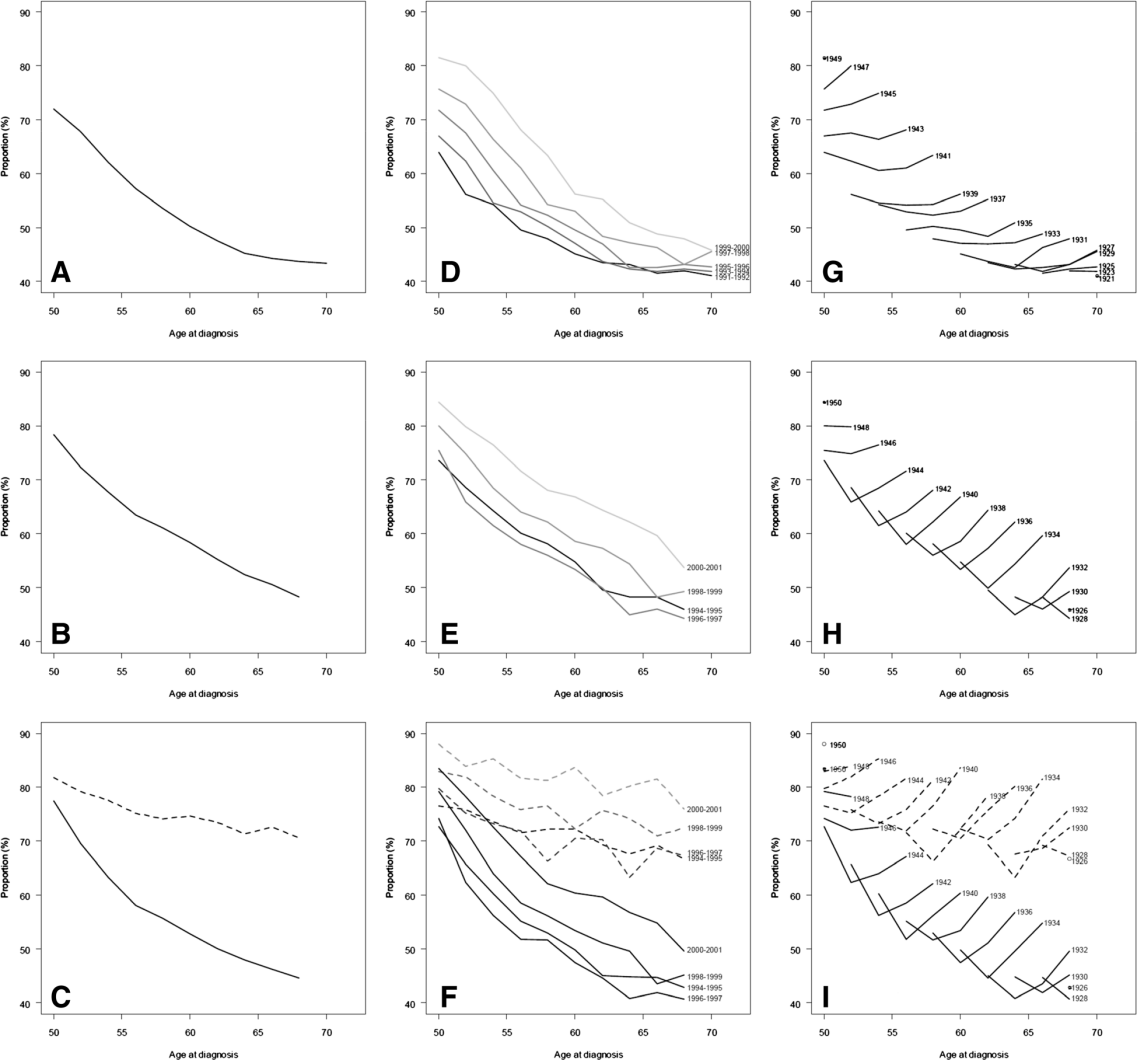
**Proportion of mixed/dense mammographic density by age (age at diagnosis), period, and birth cohort.** Mammography screening program of Copenhagen (1991–2001) and Funen (1994–2001), Denmark. Copenhagen (1991–2001): **A)** Proportion of mixed/dense mammographic density by age group, Copenhagen 1991–2001. **D)** Proportion of mixed/dense mammographic density by age group and period, Copenhagen 1991–2001. **G)** Proportion of mixed/dense mammographic density by age group and birth cohort, Copenhagen 1991–2001. Funen (1994–2001): **B)** Proportion of mixed/dense mammographic density by age group, Funen 1994–2001. **E)** Proportion of mixed/dense mammographic density by age group and period, Funen 1994–2001. **H)** Proportion of mixed/dense mammographic density by age group and birth cohort, Funen 1994–2001. Current and never hormone users, Funen (1994–2001): **C)** Proportion of mixed/dense mammographic density by age group, Funen 1994–2001. **F)** Proportion of mixed/dense mammographic density by age group and period, Funen 1994–2001. **I)** Proportion of mixed/dense mammographic density by age group and birth cohort, Funen 1994–2001. Legend: Dotted line; current users, solid line; never users.

The proportion of women with mixed/dense mammographic density decreased with increasing age in Copenhagen and Funen (Figure 1A and 1B). This pattern was seen for never and current hormone users, although the decrease was smaller among current than never users (Figure 1C). In both screening programs, the pattern across age was the same in all screening periods with a higher proportion of mixed/dense mammographic density at a given age in the later compared with the earlier screening periods (Figure 1D and 1E).The decrease in the proportion of women with mixed/dense mammographic density with increasing age was smaller among current than never hormone users (Figure 1F).

The proportions of women with mixed/dense mammographic density for each birth cohort in Copenhagen are found in the diagonals of Table 2, where each step-wise diagonal chain of squares represents a birth cohort. In Table 2, women born in 1940–1942 were 50–51 years of age in 1991–1993, 52–53 years in 1993–1995, 54–55 years in 1995–1997, 56–57 years in 1997–1999, and 58–59 years in 1999–2001. This birth cohort had a relatively constant probability of mixed/dense mammographic density at 64% in 1991–1993, 62% in 1993–1995, 61% in 1995–1997, 61% in 1997–1999, and 63% in 1999–2001. The birth cohort of 1930–1932 was 60–61 years of age in 1991–1993, 62–63 years in 1993–1995, 64–65 years in 1995–1997, 66–67 years in 1997–1999, and 68–69 years in 1999–2001. The probability of mixed/dense mammographic density in this birth cohort was stable at 45% in 1991–1993, 44% in 1993–1995, 43% in 1995–1997, 46% in 1997–1999, and 48% in 1999–2001. The probability of mixed/dense mammographic density was thus lower in the birth cohorts of 1930–1932 than in the birth cohorts of 1940–1942 at all ages. This pattern was consistent across birth cohorts among women of the same age (Figure 1G and 1H).

**Table 2:** Proportion (%) of women with mixed/dense mammographic density by screening age, period, and birth cohort

**Copenhagen**	**Period 1991**–**93 (n=29991)**	**Period 1993–95 (n=25920)**	**Period 1995–97 (n=24981)**	**Period 1997–99 (n=25269)**	**Period 1999–2001 (n=24910)**	**Total**
**Age (years)**						
50-51, n=7818	64 (63–65)	67 (66–68)	72 (71–73)	76 (75–77)	81 (81–82)	**72 (72–73)**
52-53, n=16808	56 (55–57)	62 (61–63)	68 (67–68)	73 (72–73)	80 (79–80)	**68 (68–69)**
54-55, n=14962	54 (53–55)	54 (53–55)	61 (60–61)	66 (66–67)	75 (74–75)	**63 (62–63)**
56-57, n=13546	50 (49–50)	53 (52–54)	54 (53–55)	61 (60–62)	68 (67–69)	**57 (57–58)**
58-59, n=12455	48 (47–49)	50 (49–51)	52 (51–53)	54 (53–55)	63 (62–64)	**54 (53–54)**
60-61, n=12007	45 (44–46)	47 (46–48)	50 (49–51)	53 (52–54)	56 (55–57)	**50 (49–50)**
62-63, n=11758	43 (43–44)	44 (43–45)	47 (46–48)	48 (47–49)	55 (52–54)	**47 (46–47)**
64-65, n=11627	43 (42–44)	42 (41–43)	43 (42–44)	47 (46–48)	51 (50–52)	**45 (44–45)**
66-67, n=11886	42 (41–42)	42 (41–43)	43 (42–44)	46 (45–47)	49 (48–50)	**44 (43–44)**
68-69, n=12009	42 (41–43)	42 (41–43)	43 (42–44)	43 (42–44)	48 (47–49)	**43 (43–44)**
70-71, n=6194	41 (40–42)	42 (41–43)	43 (41–44)	45 (44–47)	46 (44–47)	**43 (42–44)**
**Total**	**47 (47–48)**	**49 (49–50)**	**53 (53–54)**	**58 (57–58)**	**64 (64–64)**	
**Funen**^**1**^	**Period 1994–95 (n=39059)**	**Period 1996–97 (n=42767)**	**Period 1998–99 (n=43564)**	**Period 2000–01 (n=45275)**		**Total**
**Age (years)**						
50-51, n=23122	73 (72–74)	75 (74–76)	80 (79–81)	84 (83–85)		**78 (77–78)**
52-53, n=22277	68 (67–70)	65 (64–66)	74 (73–75)	79 (78–80)		**72 (71–73)**
54-55, n=20506	64 (63–66)	61 (60–63)	68 (66–69)	76 (74–77)		**68 (67–68)**
56-57, n=18539	60 (59–62)	58 (56–59)	64 (62–65)	71 (69–72)		**64 (63–64)**
58-59, n=16776	58 (56–59)	56 (54–47)	62 (61–64)	68 (67–69)		**61 (60–62)**
60-61, n=15664	55 (53–56)	53 (52–55)	59 (57–60)	67 (65–68)		**59 (58–59)**
62-63, n=14734	50 (49–52)	50 (49–52)	57 (55–59)	64 (63–66)		**56 (55–57)**
64-65, n=13820	49 (47–51)	45 (44–47)	54 (53–56)	62 (60–64)		**53 (52–54)**
66-67, n=13065	49 (47–51)	47 (45–49)	49 (47–51)	60 (58–62)		**51 (50–52)**
68-69, n=12162	46 (45–48)	45 (44–47)	50 (48–52)	55 (53–57)		**49 (48–50)**
**Total**	**59 (58–59)**	**58 (57–58)**	**64 (63–64)**	**70 (70–71)**		

Mammography screening program of Copenhagen (1991–2001) and Funen (1994–2001), Denmark.

^1^The second round of Funen was to some extent affected by film problems during the year 1996 which could result in some inconsistency when comparing screening round 2 with the other screening rounds.

Figure 1G shows the proportion of women with mixed/dense mammographic density by age and birth cohort in Copenhagen. For each birth cohort, the proportion of women with mixed/dense mammographic density was stable across age. However, the proportion of women with mixed/dense mammographic density increased from 40-45% in women born in the 1920s to 75-80% in women born in the late 1940s. Although we did not have data for all age groups from these two birth cohorts it is unlikely that the graphs would have overlapped. For nearly all birth cohorts, the proportion of women with mixed/dense mammographic density was slightly higher at the last observed point.

Figure 1H shows the proportion of women with mixed/dense mammographic density by age group and birth cohort in Funen. The proportion of women with mixed/dense mammographic density was lower in the 2^nd^ period and higher in the 4^th^ period, than in the 1^st^ and 3^rd^ periods. The proportion of women with mixed/dense mammographic density in Funen increased from 45% among women born in the late 1920s to 75-80% among women born in the late 1940s.

Figure 1I and Table 3 show the proportion of women with mixed/dense mammographic density by age, birth cohort, and hormone use in Funen. The proportion of never users with mixed/dense mammographic density was similar to the proportion of all women with mixed/dense mammographic density, increasing from 45% among women born in the late 1920s to 75-80% among women born in the late 1940s. The proportion of current users with mixed/dense mammographic density increased from around 70% for women born in the late 1920s to 80-85% for women born in the late 1940s. The difference in the proportion of women with mixed/dense mammographic density found between never and current users born in the late 1920s (45% versus 71%) had clearly decreased for those born in the late 1940s (62% versus 75%). Sensitivity analysis restricted to combined estrogen-progestogen users showed similar results (data not shown).

**Table 3:** Proportion (%) of women with mixed/dense mammographic density by hormone use (HT), screening age, period, and birth cohort

**Never HT users**	**Period 1994–95 (n=26916)**	**Period 1996–97 (n=28065)**	**Period 1998–99 (n=27306)**	**Period 2000–01 (n=27315)**	**Total**
**Age (years)**					
50-51, n=11858	73 (72–73)	74 (74–75)	79 (79–80)	83 (82–85)	**77 (77–78)**
52-53, n=10006	66 (65–66)	62 (61–63)	72 (71–73)	78 (77–80)	**70 (69–71)**
54-55, n=8378	60 (59–61)	56 (55–57)	64 (63–65)	73 (71–74)	**64 (63–65)**
56-57, n=7599	55 (54–56)	52 (51–53)	59 (57–59)	67 (65–69)	**58 (58–59)**
58-59, n=7298	53 (52–54)	52 (51–53)	56 (55–57)	62 (60–64)	**56 (55–57)**
60-61, n=7468	50 (49–51)	47 (46–48)	53 (52–54)	60 (58–62)	**53 (52–54)**
62-63, n=7422	45 (44–46)	45 (44–46)	51 (50–52)	60 (58–62)	**50 (49–51)**
64-65, n=7556	45 (44–46)	41 (40–42)	50 (49–51)	57 (55–59)	**48 (47–49)**
66-67, n=7475	45 (44–46)	42 (41–43)	43 (42–44)	55 (53–57)	**46 (45–47)**
68-69, n=7227	43 (42–44)	41 (40–42)	45 (44–46)	50 (48–52)	**44 (43–45)**
**Total**	**54 (54–55)**	**53 (53–54)**	**59 (59–60)**	**66 (66–67)**	
**Current HT users**	**Period 1994–95 (n=8171)**	**Period 1996–97 (n=9040)**	**Period 1998–99 (n=9464)**	**Period 2000–01 (n=9669)**	**Total**
**Age (years)**					
50-51, n=3649	76 (75–77)	80 (79–81)	83 (82–84)	88 (86–90)	**81 (80–83)**
52-53, n=4025	76 (75–77)	75 (74–76)	82 (81–83)	84 (82–86)	**79 (78–80)**
54-55, n=3788	73 (72–75)	73 (72–74)	78 (77–79)	85 (83–87)	**78 (77–79)**
56-57, n=3462	71 (70–73)	72 (71–73)	76 (75–77)	82 (80–84)	**75 (74–77)**
58-59, n=2986	72 (71–73)	66 (65–67)	76 (75–78)	81 (79–84)	**74 (73–76)**
60-61, n=2511	73 (71–74)	70 (69–72)	72 (71–73)	83 (81–86)	**75 (73–76)**
62-63, n=2047	69 (68–71)	71 (69–72)	75 (74–77)	78 (76–81)	**74 (72–75)**
64-65, n=1631	68 (66–69)	63 (61–65)	74 (73–76)	80 (77–83)	**72 (70–74)**
66-67, n=1414	69 (67–71)	69 (68–70)	71 (69–73)	82 (78–85)	**73 (71–75)**
68-69, n=1162	66 (64–69)	67 (65–69)	72 (70–74)	76 (72–80)	**70 (68–73)**
**Total**	**73 (72–73)**	**72 (71–73)**	**77 (76–78)**	**83 (82–83)**	

Mammography screening program of Funen (1994–2001), Denmark.

Table 4 shows odds ratios for the association between risk of mixed/dense mammographic density and birth cohort, screening age, and screening period. In Copenhagen, the age-adjusted OR (95% CI) of mixed/dense mammographic density in women born in 1941–42 and 1949–50 was 2.48 (2.22-2.76) and 5.41 (4.22-6.94), respectively, compared with women born in the years 1921–22. In Funen, the age-adjusted OR of mixed/dense mammographic density in women born in 1946–47 was 5.89 (5.32-6.51) compared with women born in 1924–25. Mutually linear adjustments did not alter the results in the multivariate analysis (data not shown). Although age was a significant predictor of mixed/dense mammographic density, much smaller effect sizes for age were seen than for birth cohort. Sensitivity analysis in subgroups of women with mammographic density assessments conducted within the same screening period confirmed the age- and birth cohort patterns found in the overall data (data not shown), and it therefore seems unlikely that the age and birth cohort effects were explained by systematic period effects and fluctuations over time in the density assessment between screening periods.

**Table 4:** Odds ratios (95% CI) of mixed/dense mammographic density by screening age, period, and birth cohort

**Copenhagen**	N F/MD^*^	**Crude odds ratio**	**Adjusted odds ratio**	**Funen**	N F/MD^*^	**Crude odds ratio**	**Adjusted odds ratio**
**Age**^**2**^				**Age**^**2**^			
50-51	2169|5649	Ref.	Ref.	50-51	5125|17997	Ref	Ref
52-53	5300|11508	0.87 (0.84-0.90)	0.92 (0.89-0.95)	52-53	6232|16045	0.82 (0.80-0.83)	0.89 (0.86-0.91)
54-55	5548|9414	0.78 (0.76-0.81)	0.89 (0.85-0.92)	54-55	6620|13886	0.74 (0.72-0.76)	0.87 (0.85-0.90)
56-57	5763|7783	0.71 (0.69-0.74)	0.86 (0.82-0.90)	56-57	6749|11790	0.68 (0.66-0.70)	0.88 (0.85-0.91)
58-59	5783|6672	0.67 (0.64-0.69)	0.86 (0.82-0.90)	58-59	6513|10263	0.64 (0.62-0.66)	0.92 (0.89-0.95)
60-61	6015|5992	0.62 (0.60-0.65)	0.85 (0.81-0.90)	60-61	6501|9163	0.60 (0.58-0.62)	0.97 (0.93-1.02)
62-63	6225|5533	0.59 (0.57-0.62)	0.86 (0.82-0.90)	62-63	6519|8215	0.58 (0.56-0.60)	1.05 (1.00-1.10)
64-65	6408|5219	0.55 (0.53-0.58)	0.85 (0.80-0.89)	64-65	6514|7306	0.56 (0.54-0.58)	1.12 (1.07-1.17)
66-67	6685|5201	0.54 (0.52-0.56)	0.86 (0.81-0.91)	66-67	6360|6705	0.56 (0.54-0.58)	1.22 (1.16-1.29)
68-69	6798|5211	0.53 (0.51-0.55)	0.88 (0.83-0.93)	68-69	6183|5979	0.54 (0.52-0.56)	1.30 (1.23-1.37)
70-71	3532|2662	0.50 (0.48-0.53)	0.87 (0.81-0.92)	-	-	-	-
**Period**^**2**^				**Period**^**2**^			
1991-1993	15814|14177	Ref.	Ref.	1994-1995	16100|22959	Ref	Ref
1993-1995	13092|12827	0.98 (0.96-1.00)	0.95 (0.93-0.96)	1996-1997	18031|24736	0.84 (0.83-0.86)	0.81 (0.80-0.82)
1995-1997	11668|13313	1.00 (0.98-1.02)	0.94 (0.92-0.96)	1998-1999	15733|27831	0.97 (0.96-0.99)	0.91 (0.90-0.93)
1997-1999	10669|14600	1.01 (0.99-1.04)	0.92 (0.90-0.94)	2000-2001	13452|31823	1.15 (1.13-1.18)	1.05 (1.03-1.07)
1999-2001	8983|15927	1.11 (1.08-1.13)	0.97 (0.95-1.00)	-	-	-	-
**Birthcohort**^**1**^				**Birthcohort**^**1**^			
1921-1922	1599|1117	Ref.	Ref.	1924-1925	1620|1401	Ref.	Ref.
1923-1924	3276|2334	1.02 (0.93-1.13)	1.02 (0.93-1.13)	1926-1927	3359|3008	1.01 (0.93-1.11)	1.04 (0.96-1.14)
1925-1926	4508|3258	1.04 (0.94-1.15)	1.05 (0.95-1.16)	1928-1929	4972|4721	1.10 (1.01-1.20)	1.19 (1.09-1.30)
1927-1928	5285|4026	1.11 (1.00-1.22)	1.12 (1.01-1.24)	1930-1931	6579|6518	1.21 (1.11-1.32)	1.35 (1.24-1.48)
1929-1930	6047|4746	1.13 (1.03-1.25)	1.15 (1.04-1.28)	1932-1933	6323|7672	1.42 (1.30-1.54)	1.70 (1.55-1.86)
1931-1932	6166|5315	1.26 (1.14-1.39)	1.28 (1.15-1.42)	1934-1935	6415|8665	1.54 (1.42-1.68)	1.98 (1.81-2.17)
1933-1934	5880|5270	1.32 (1.20-1.46)	1.35 (1.21-1.50)	1936-1937	6573|9729	1.77 (1.63-1.92)	2.40 (2.19-2.63)
1935-1936	5705|5888	1.51 (1.36-1.66)	1.53 (1.37-1.70)	1938-1939	6529|10507	1.99 (1.83-2.17)	2.83 (2.58-3.11)
1937-1938	5547|6556	1.71 (1.55-1.89)	1.71 (1.53-1.90)	1940-1941	6588|12407	2.20 (2.03-2.39)	3.18 (2.90-3.50)
1939-1940	5716|7490	1.91 (1.73-2.10)	1.86 (1.67-2.07)	1942-1943	6638|14842	2.76 (2.55-3.00)	3.90 (3.54-4.28)
1941-1942	4300|7531	2.56 (2.32-2.82)	2.48 (2.22-2.76)	1944-1945	4686|13950	3.27 (3.01-3.55)	4.63 (4.21-5.10)
1943-1944	3270|7371	3.27 (2.97-3.61)	3.12 (2.80-3.48)	1946-1947	2429|9351	4.23 (3.88-4.61)	5.89 (5.32-6.51)
1945-1946	2032|6151	4.40 (3.98-4.87)	4.15 (3.71-4.64)	1948-1949	875|4578	5.93 (5.37-6.55)	7.68 (6.86-8.61)
1947-1948	805|3400	6.21 (5.55-6.95)	5.72 (5.05-6.47)	-	-	-	-
1949-1950	90|391	6.22 (4.88-7.92)	5.41 (4.22-6.94)	-	-	-	-

Mammography screening program of Copenhagen (1991–2001) and Funen (1994–2001), Denmark.

^*^F/MD: F=fatty mammographic density; MD=mixed/dense mammographic density.

^1^Adjusted for screening age.

^2^Adjusted for birth cohort.

## Discussion

This study on data from two independent screening programs showed an increased risk of mixed/dense mammographic density across birth cohorts. Within a given birth cohort, the probability of having mixed/dense mammographic density remained fairly stable across age. These findings have to our knowledge not been explored in previous studies.

The Danish population is homogeneous with most women being Caucasian. Since data for this study derived from comprehensive registers recall and reporting biases were unlikely. A limitation of the study was the qualitative dichotomous measure for mammographic density available defined as mixed/dense relative to fatty mammographic density by senior radiologists. Since qualitative measures tend to overestimate the degree of density and are less precise than quantitative measures of mammographic density [23], subtle changes in mammographic density in the age-period-cohort modeling could potentially be masked. To ensure consistency in the density assessment, the analysis was restricted to a study period where the same radiologists by consensus reading were in charge of the density assessment. 14% and 24% of women in Copenhagen and Funen, respectively, changed mammographic density category over time within the 10-years of follow-up in our study. The 10% difference between the two programs might be explained by a known larger proportion of women in the ages of menopausal transition 50–54 years in the Funen compared to the Copenhagen mammographic screening program [24]. The proportions of women with changed mammographic density category in Copenhagen and Funen were as expected considering the 10-years follow-up period. Record linkage by the unique personal identification numbers ensured correct linkage of records. We find it unlikely that selection bias affected our results since coverage differed between the two programs with similar results. However, we did not have information available on body mass index (BMI) for the whole cohort and systematic bias could have occurred, if obese women from younger birth cohorts were more likely to abstain from mammography screening than obese women from older birth cohorts, since obesity is inversely associated with mammographic density [25]. In a subanalysis of 5134 women with available data on objectively measured BMI from the Diet, Cancer, and Health cohort study [26], we found no systematic differences across birth cohorts among obese women abstaining from mammography screening (data not shown). The association between non-participation and women’s educational level or socio-economic status was found to be U-shaped with a strong urban–rural gradient in non-participation [24,27,28]. The age-period-cohort modeling in Table 4 was not adjusted for reproductive or life style risk factors pertaining to women’s lives, since we did not have information available for the whole cohort, wherefore residual confounding cannot be completely ruled out.

The birth cohort adjusted odds of mixed/dense breasts across period remained steady in Copenhagen but increased in Funen (Table 4). This period effect in Funen was likely caused by a lower percentage of women with mixed/dense mammographic density in the 2^nd^ invitation round due to temporary film quality problems in 1996 in Funen [18]. In both programs we found elevated proportions of women with mixed/dense mammographic density in the latest invitation rounds. From 2000 to 2004, two projections of the breasts were used increasingly and became standard in 2004. Since two projections encoded mixed/dense mammographic density, a concern could be if this drift seen for all ages during the last screening round could compensate for a biologically true decline in mammographic density over age. We therefore restricted the analysis to the period 1991–2001 where no systematic changes in screening practice or mammographic density assessment took place in the two programs, to reduce the probability that systematic changes in screening practice could have caused the findings attributed to the age- and birth cohort effects.

Boyd et al. [11] distinguished between cross-sectional studies, where mammographic features of different women are compared, and longitudinal studies on changes in mammographic density of individual women. Though birth cohort trends in women’s mammographic density have not been investigated in previous studies, the largest study on longitudinal measurements from the Breast Cancer Surveillance Consortium [29] did report a BI-RADS density distribution across age-groups consistent with the birth cohort effects found in our study. The Breast Cancer Surveillance Consortium covered 301,955 women aged 30 to 70+ years. The follow-up time was for half of the women less than three years from the first to the last mammogram. Overall, there was no change in the women’s distribution by BI-RADS density codes between the first and last mammogram, though the short follow-up time should be taken into account. There was, however, a considerable difference across age groups consistent with an increase in mammographic density by year of birth. The proportion of BI-RADS density code 3 or 4 at last mammogram was 64% in women aged 30–39 years, 60% aged 40–49, 40% aged 50–59, 29% aged 60–69, and 29% in women aged 70+ years. The decrease from the age group 40–49 to 50–59 was consistent with a decrease in mammographic density related to changing menopausal status.

Boyd et al. [11] found a 5% decrease in mammographic density among women premenopausal at first and postmenopausal at next mammography in a study population of 202 women, with an excess decrease in mammographic density of 3% compared with an age-matched group of women remaining premenopausal. McCormack et al. [30] found a decreased median mammographic density from 20% at first to 12% at third screening among 226 women aged 50–52 years at recruitment, and a decrease from 15% to 11% in 155 women aged 53–65 years at recruitment. Verheus et al. [31] followed 684 women average aged 46.5 years with an average follow-up of 5.5 years, who were premenopausal at initial screening and postmenopausal at final screening. Mammographic density decreased during menopause from 44% at premenopausal status to 34% at postmenopausal status. These longitudinal data from screening populations unanimously showed a moderate decrease in mammographic density related to changing menopausal status and moderate changes at later age. In the Copenhagen data, we did not see a decrease in the proportion of women with mixed/dense mammographic density around menopause from age 50–51 to age 52–53 in the four birth cohorts where data were available for these age groups (Figure 1G). However, based on data reported by Danish nurses [32] more than half of Danish women were expected to be postmenopausal at their first invitation to screening. Further, it is questionable whether the dichotomous variable in mixed/dense versus fatty mammographic density would be sensitive enough to detect a moderate decrease in mammographic density during menopause. In the Funen data, the trend for ages 50–51 and 52–53 was difficult to follow due to the film problems in the second invitation round. Nevertheless, our observation of fairly stable proportions of women with mixed/dense mammographic density in the postmenopausal ages was consistent with the moderate density changes seen for postmenopausal women in other longitudinal studies.

There is a well-established positive association between hormone use and mammographic density [14-16]. We found that hormone use had a considerable impact on the proportion of women with mixed/dense mammographic density among women born in the late 1920s but less among women born in the late 1940s. This difference may be explained by a higher number of hormone related exposures in younger compared with older birth cohorts of women, among others changes in postmenopausal hormone therapy over time could have had an impact. A higher number of cohort borne breast cancer risk factors in younger compared with older birth cohorts of Danish women has previously been linked to the increasing breast cancer incidence across birth cohorts of women over time in Denmark [5]. Changes over time in native Danish women’s reproductive pattern, increasing obesity, and sedentary lifestyle could be proxies for increased sex- and growth hormones levels potentially influencing changes in women’s mammographic density [4,6].

The results of the current study must be interpreted with care and warrant future replication in larger individual level data with longer follow-up and with preferable a quantitative measure for mammographic density and adjustment for reproductive and life style covariates.

## Conclusions

Data from two large independent population-based mammography screening programs showed an increased proportion of women with mixed/dense mammographic density from 45% in birth cohorts of the late 1920s to 75-80% in birth cohorts of the late 1940s. Hormone use had a greater impact on women’s mammographic density in birth cohorts of the 1920s and less in birth cohorts of the 1940s. The birth cohort pattern and the attenuated impact of hormone use suggest that mammographic density in postmenopausal age might be linked to changing exposures over time accumulated in women’s lives.

The results must be interpreted with care and should be replicated in future studies.

## Competing interests

The authors declare that they have no competing interests.

## Authors’ contributions

All the authors have made substantial contributions to the study. SSH, SHN participated in the conception and design of the study; acquisition of the data; performed the statistical analysis; participated in analysis and interpretation of the data; drafted and finalized the manuscript. WS carried out processing and interpretation of the data on mammography screening from the Funen region; revised the manuscript critically. IV carried out processing and interpretation of the data on mammography screening from the Copenhagen region; revised the manuscript critically. EL conceived the study, participated in the design and drafting of the study; participated in analysis and interpretation of the data; revised the manuscript critically for important intellectual content and finalized the manuscript in collaboration with SSH and SHN. All authors had full access to all of the data in the study and have read and given final approval of the version to be published.

## Pre-publication history

The pre-publication history for this paper can be accessed here:

http://www.biomedcentral.com/1471-2407/13/409/prepub
